# Supplementary material for: Evaluation of the Quantitative and Structural Antimicrobial Activity of Thymol, Terpinen-4-ol, Citral, and E-2-Dodecenal, Antibiotic Molecules Derived from Essential Oils
Source: Antibiotics (Basel). 2025 Dec 1;14(12):1202. doi: 10.3390/antibiotics14121202 (PMC12729705; doi:10.3390/antibiotics14121202)
Supplement: Supplementary file 1 [file antibiotics-14-01202-s001.zip › Suplementary material 1.pdf]

E-2-Dodecenal Vs Candida tropicales an example of MIC determination

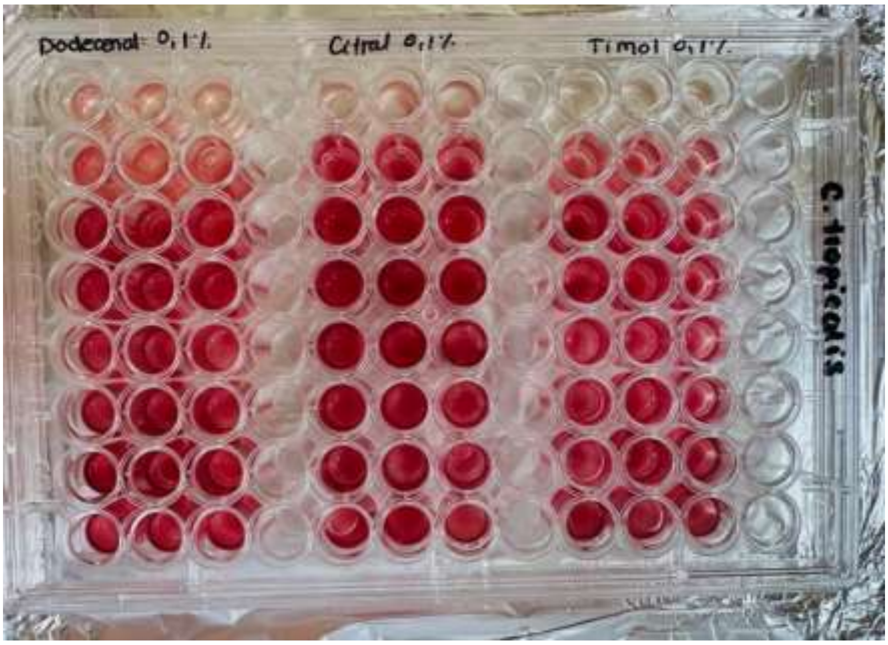

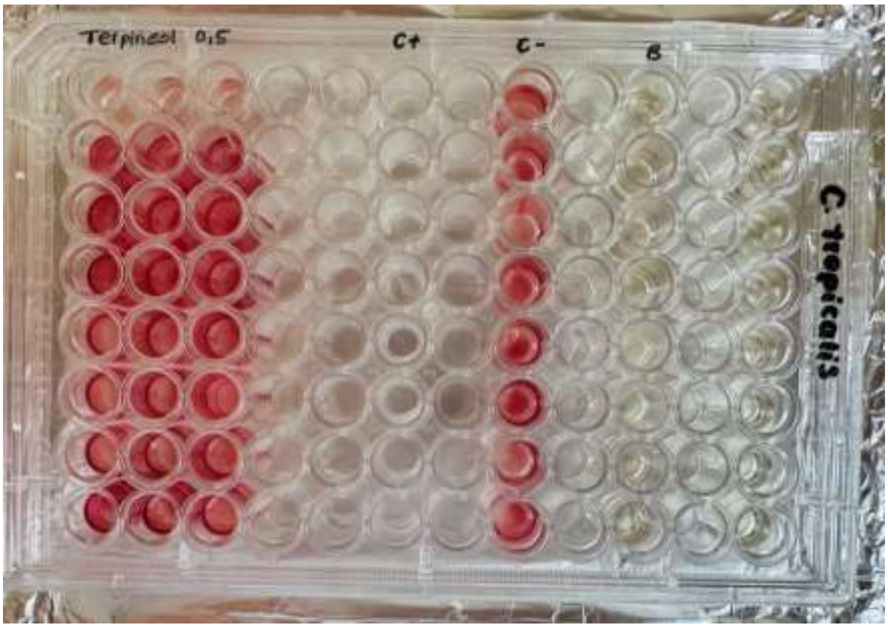

Conc ug/mL	R_1		R_2		R3	
	Abs media	% Inh	Abs muestra	% Inhibición	Abs muestra	% Inhibición
0,087	0,109	90,512	0,126	89,027	0,129	88,321
0,044	0,146	87,279	0,152	86,754	0,158	87,456
0,022	0,222	78.387	0,213	81,424	0,211	81,762
0,003	0,329	71,287	0,344	69,976	0,337	70,325

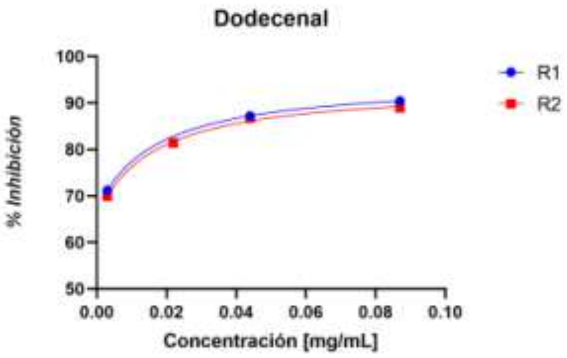

**calibration curves**	**R1**	**R2**	**R3**
	$y = 5{,}6103\ \ln(x) + 104{,}49$	$y = 17{,}867 \ln(x) + 145{,}55$	y= 13,765 ln(x) + 138,19

	**R1**	**R2**	**R3**	**media**
**MIC** [µg/mL]	80,00	75,00	73,00	76,00
