# Supplementary material for: Evaluation of the Quantitative and Structural Antimicrobial Activity of Thymol, Terpinen-4-ol, Citral, and E-2-Dodecenal, Antibiotic Molecules Derived from Essential Oils
Source: Antibiotics (Basel). 2025 Dec 1;14(12):1202. doi: 10.3390/antibiotics14121202 (PMC12729705; doi:10.3390/antibiotics14121202)

Suplementary material 2

Streptococcus mutans control

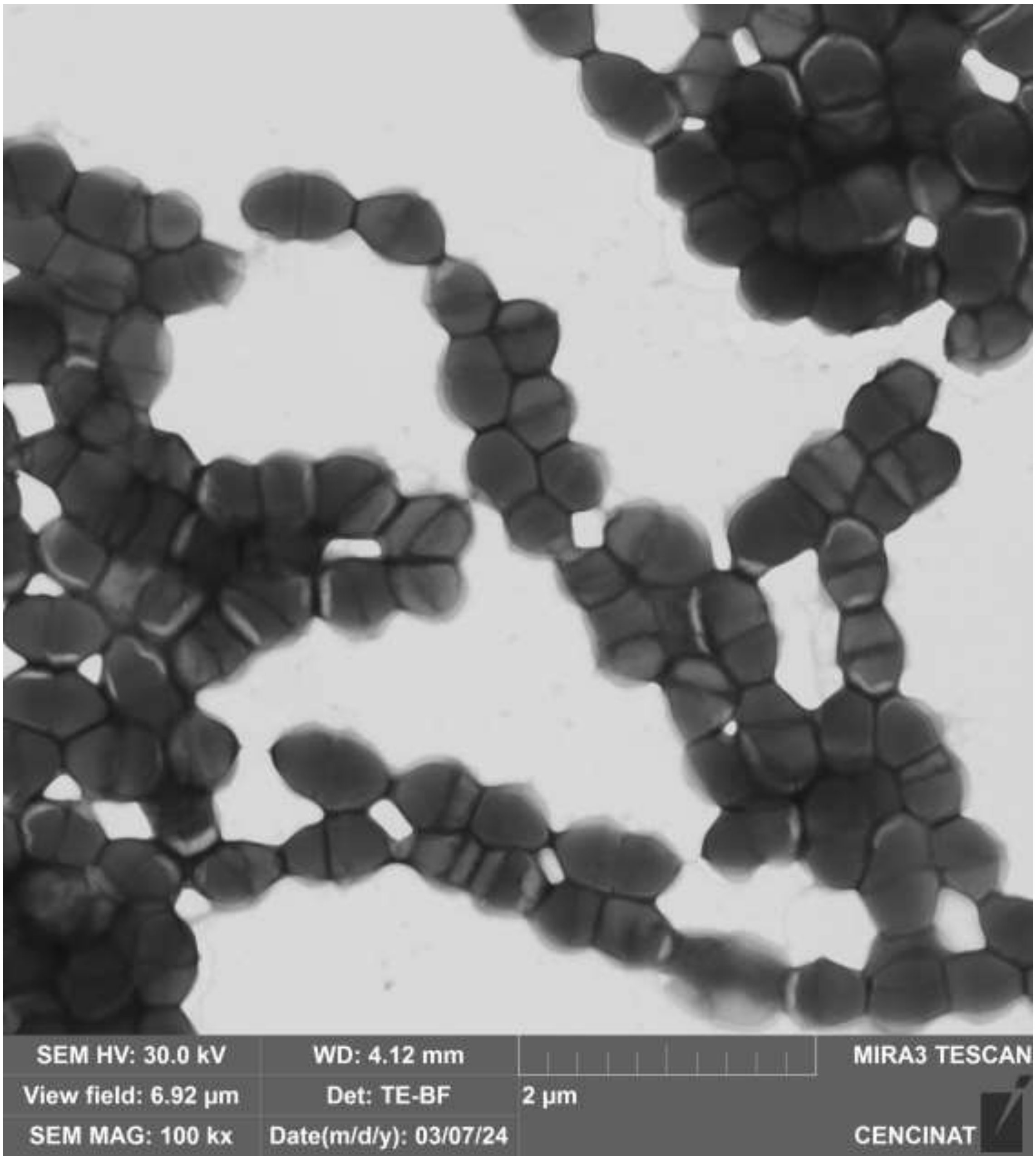

Streptococcus mutans with citral

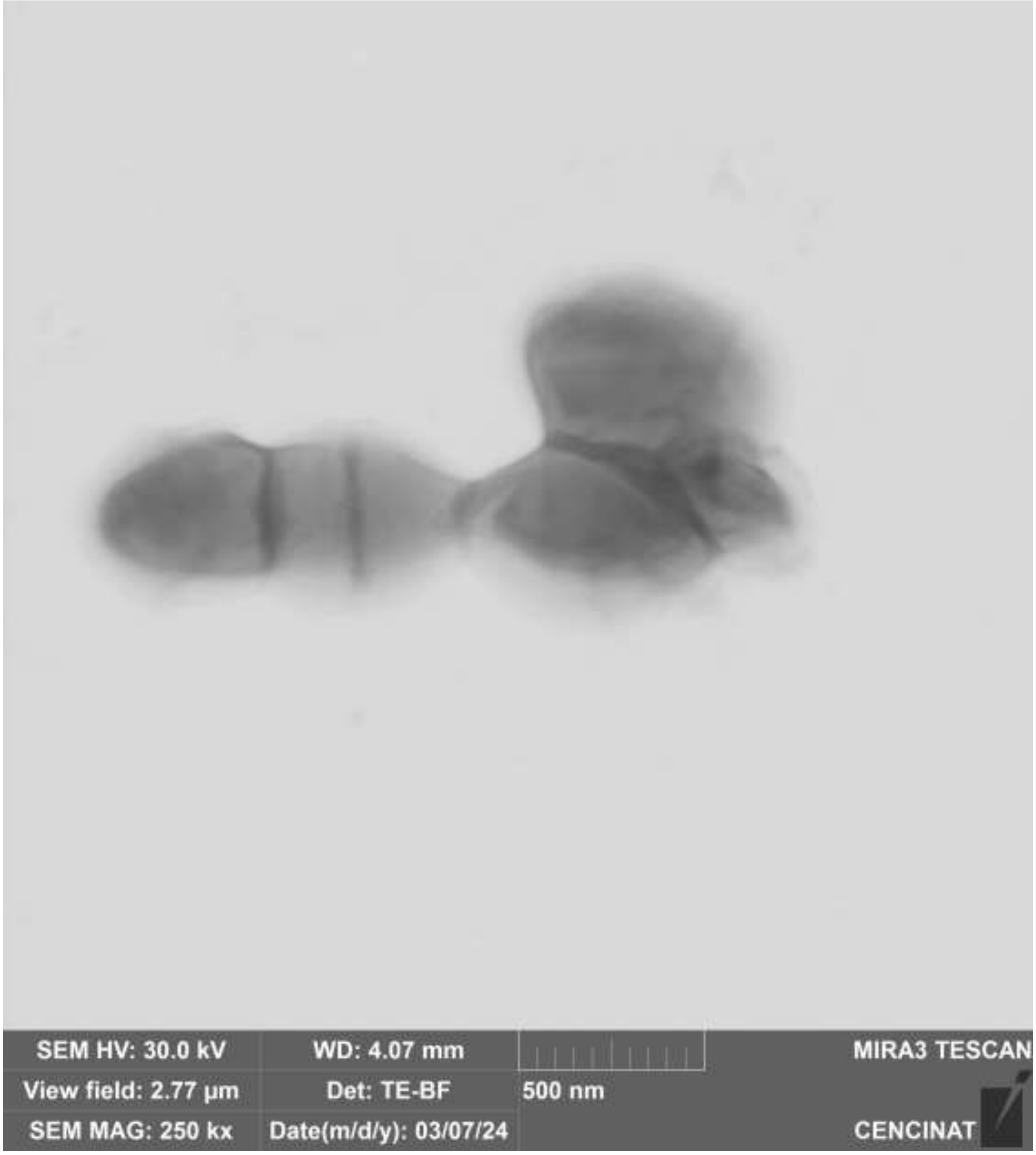

Streptococcus mutans with E-2-dodecenal

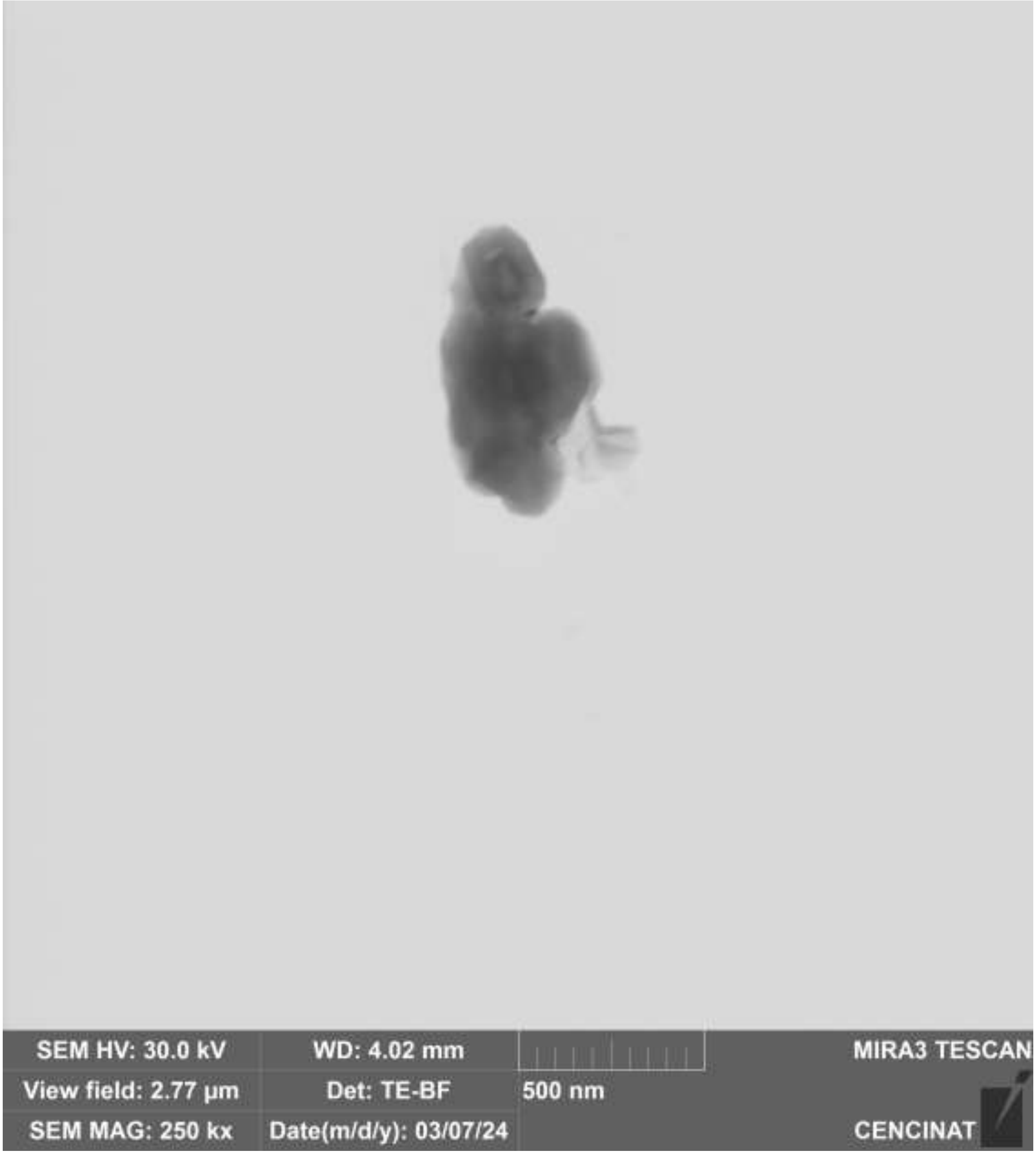

Streptococcus mutans with terpenen 4-ol

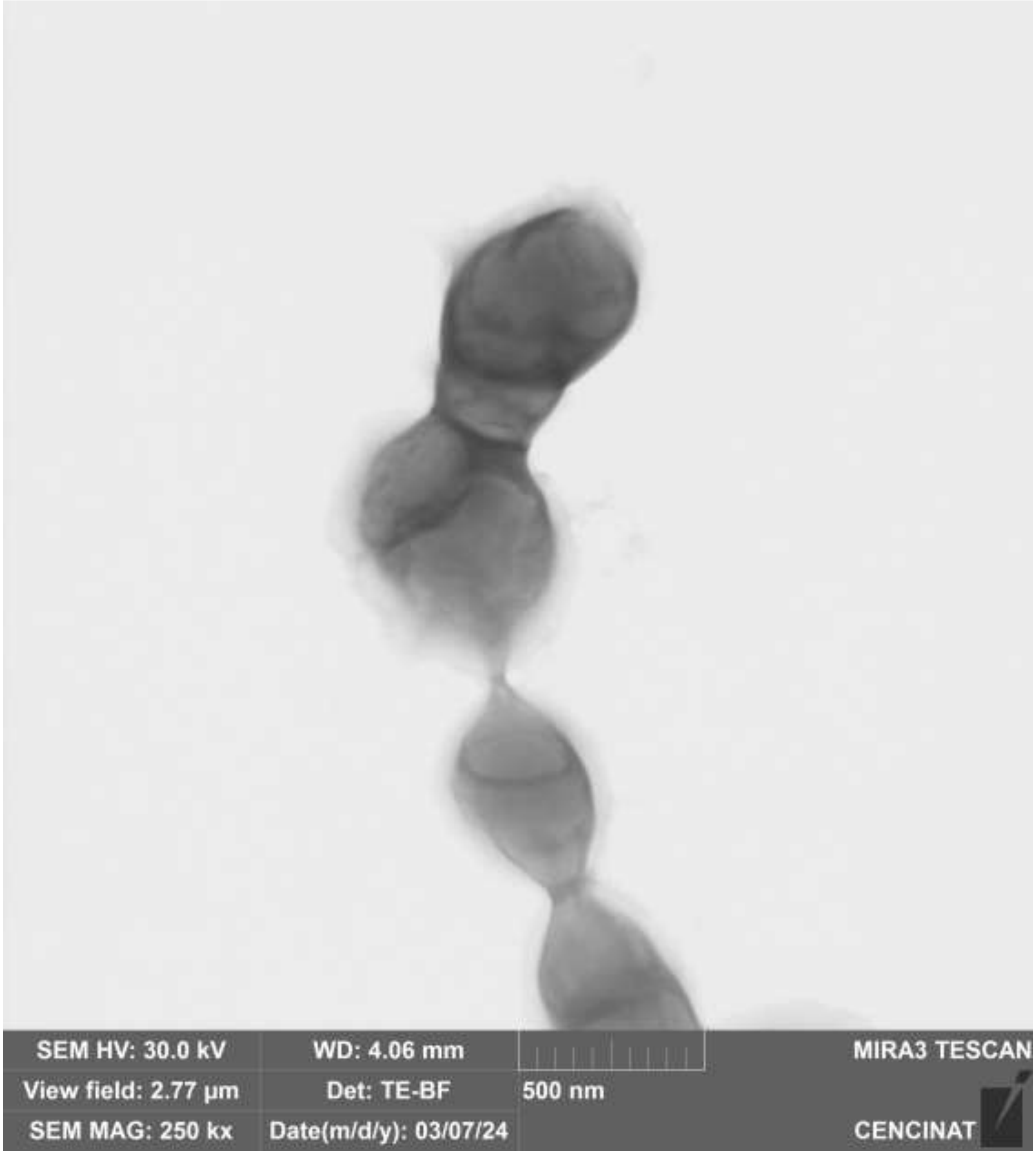

Streptococcus mutans with thymol

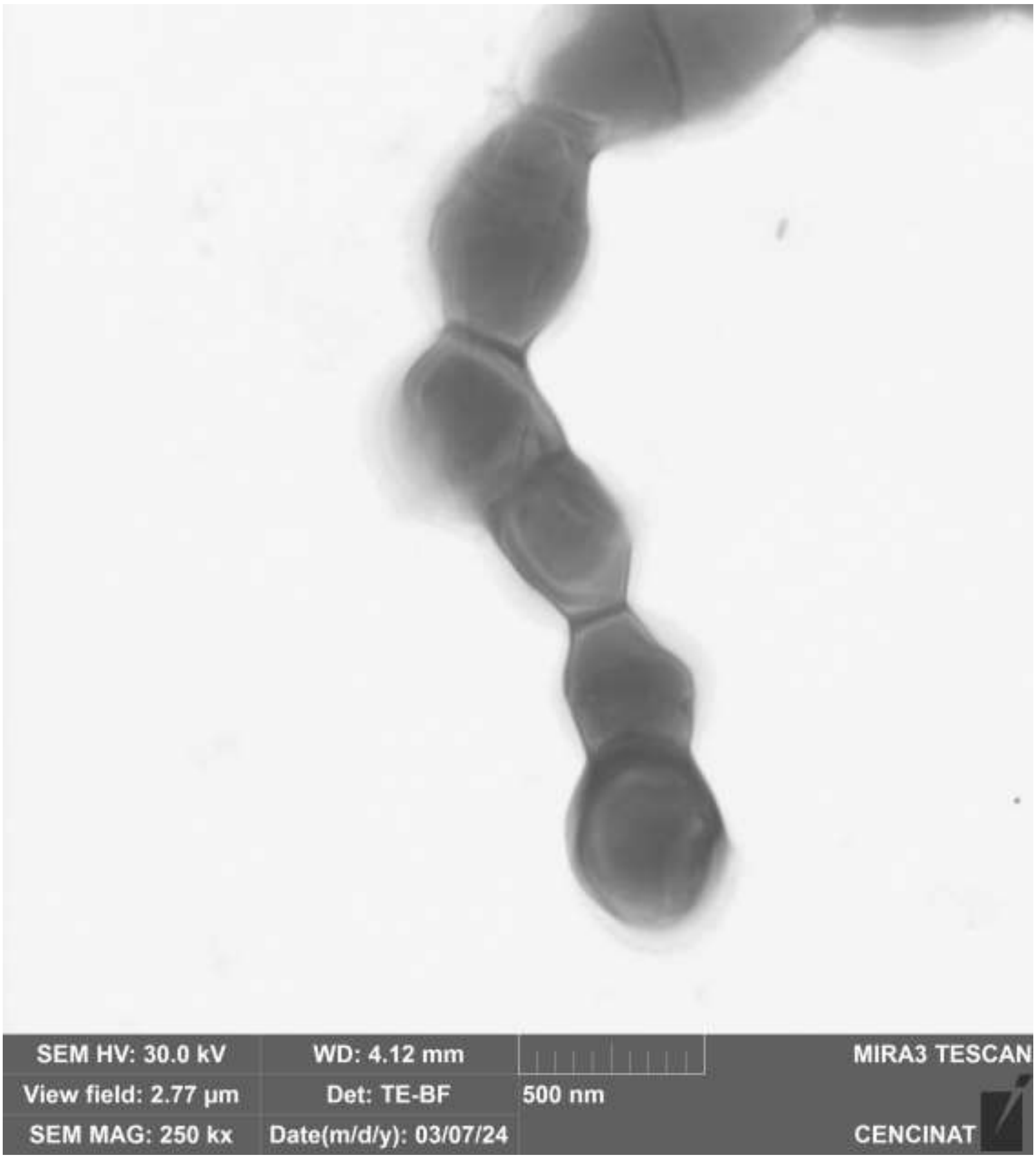

Klebsiella oxytoca control

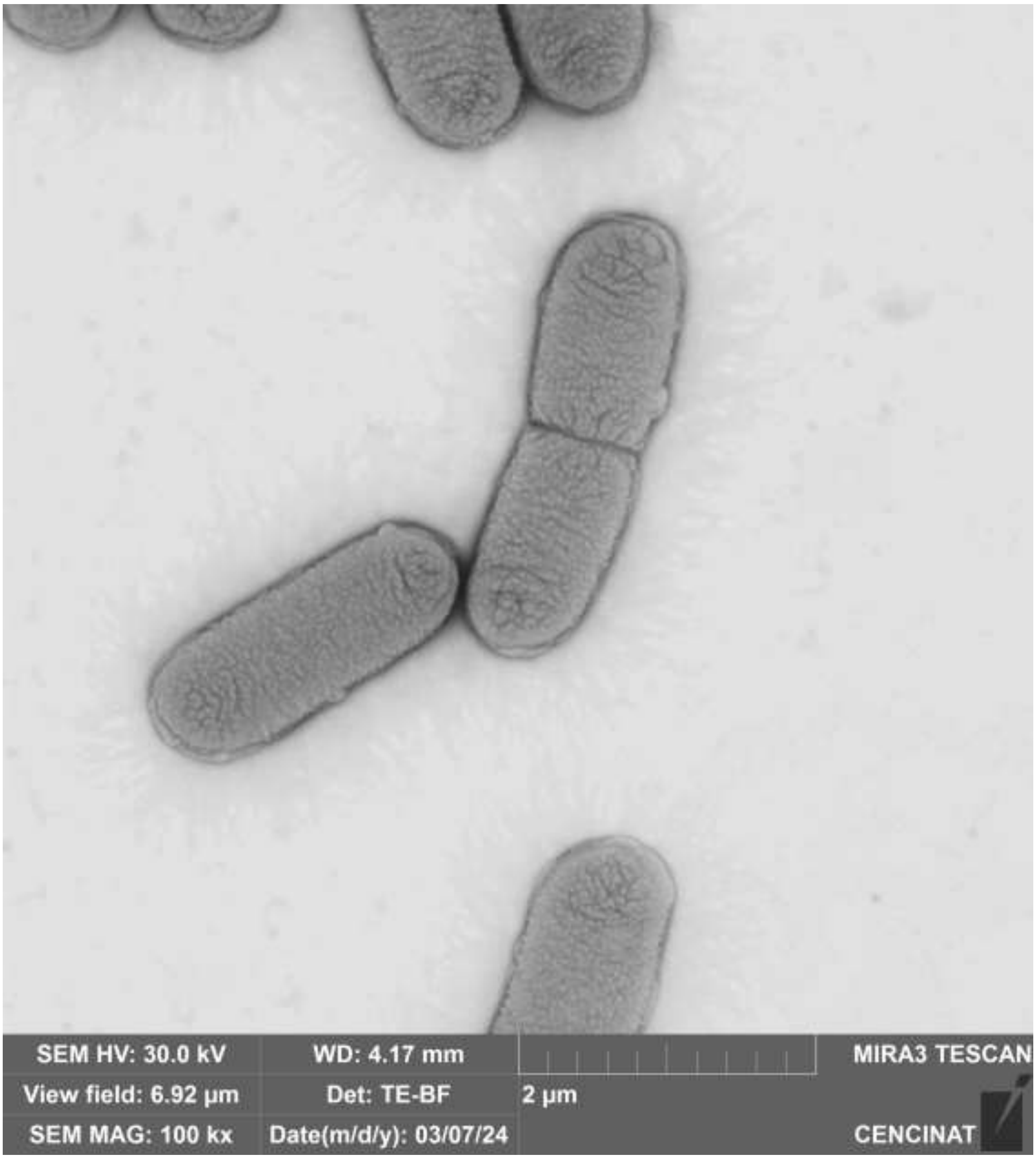

Klebsiella oxytoca with citral

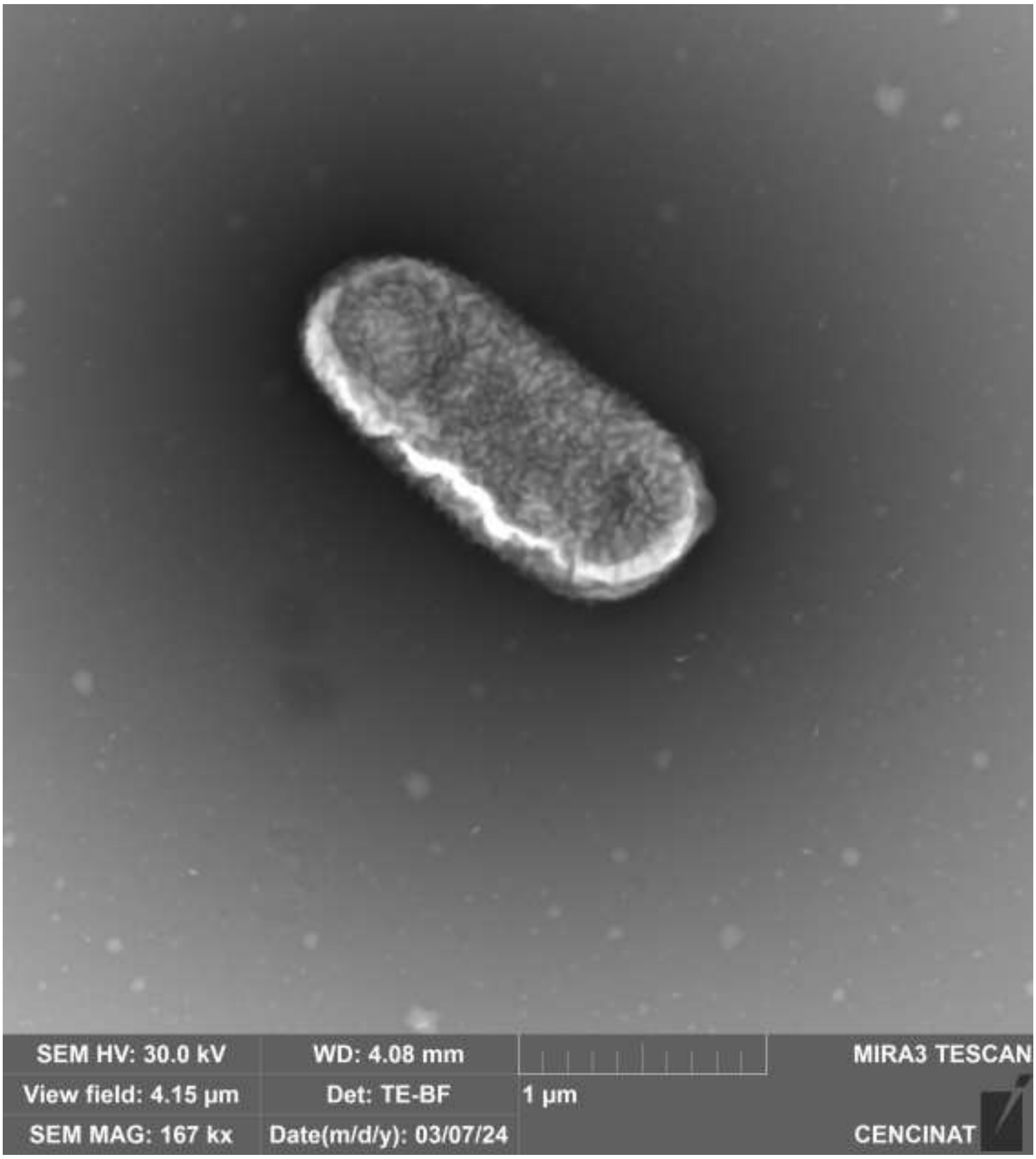

Klebsiella oxytoca with E-2-dodecenal

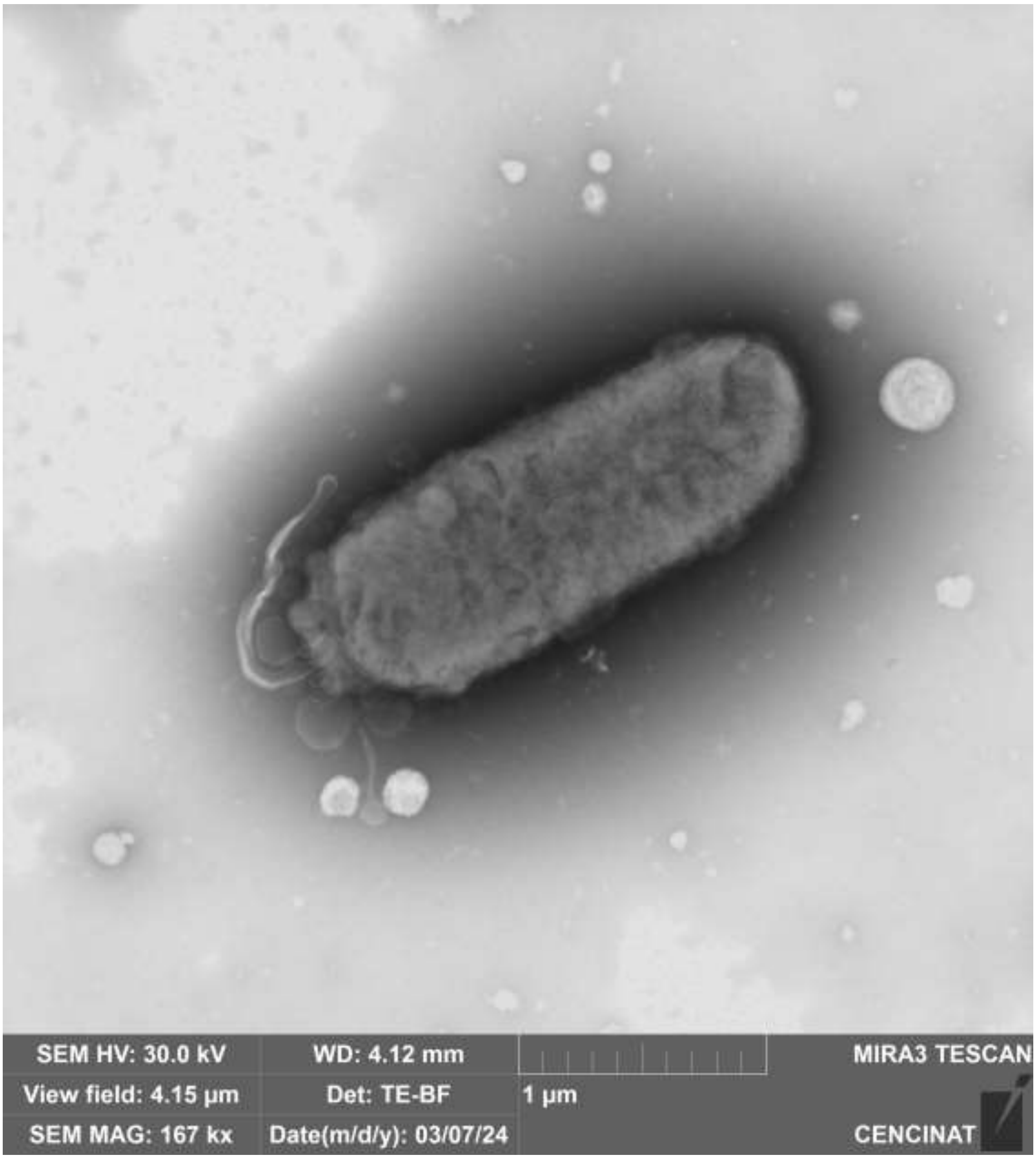

Klebsiella oxytoca with terpinen-4-ol

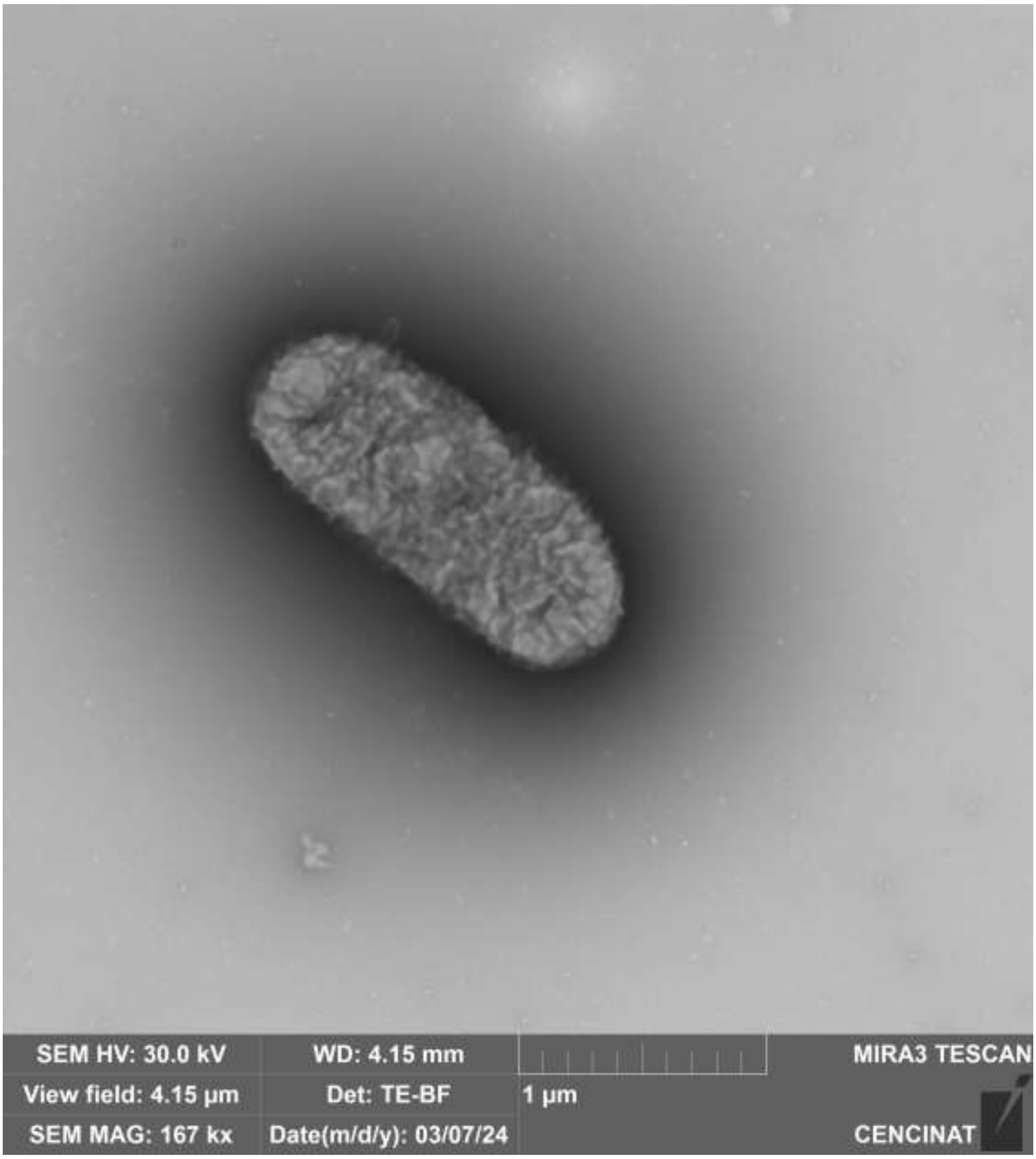

Klebsiella oxytoca with thymol

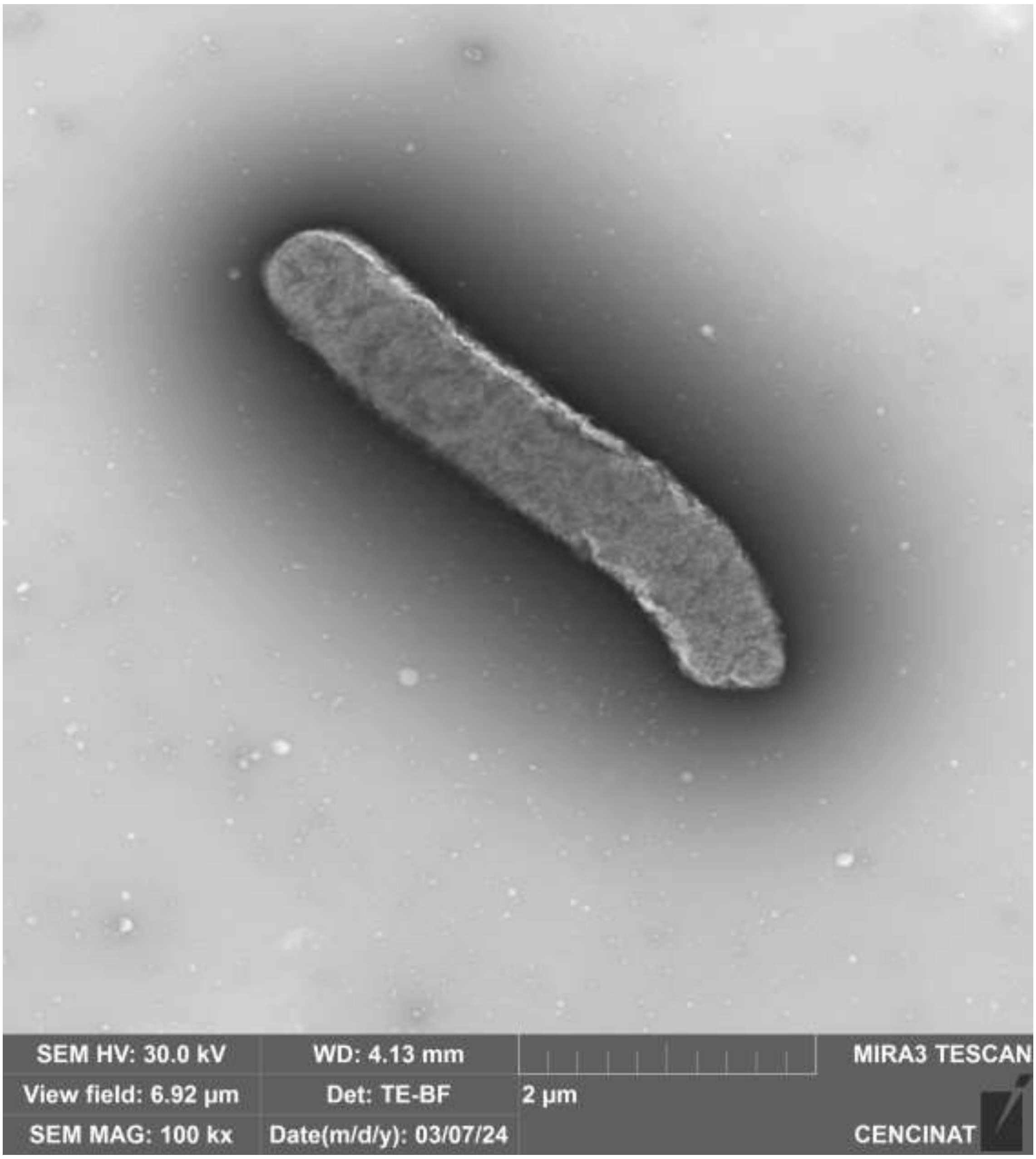

Candida tropicalis control

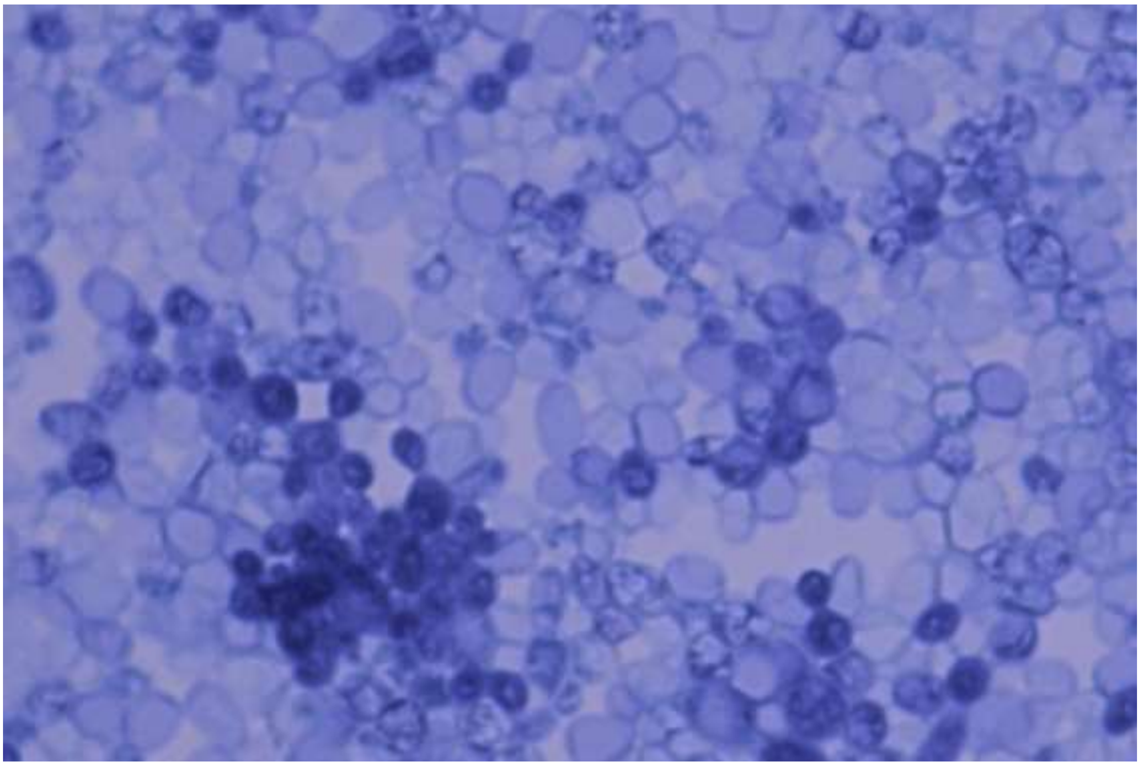

Candida tropicalis with citral

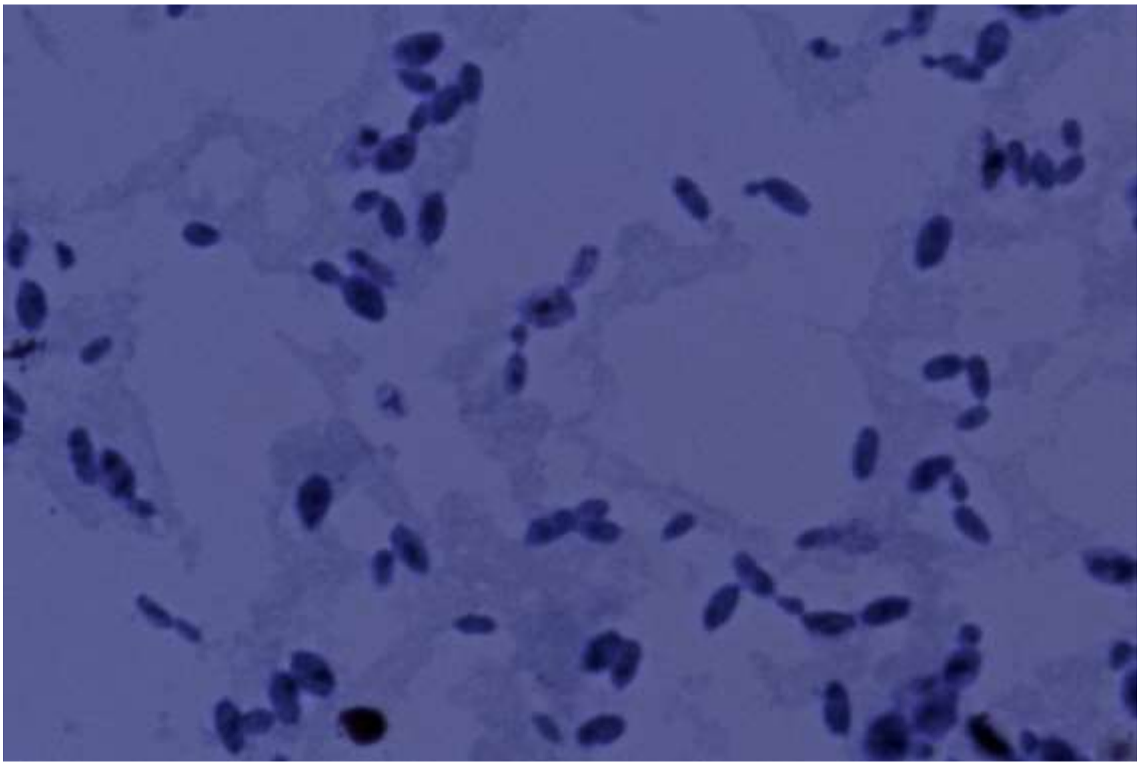

Candida tropicalis with E-2-dodecenal

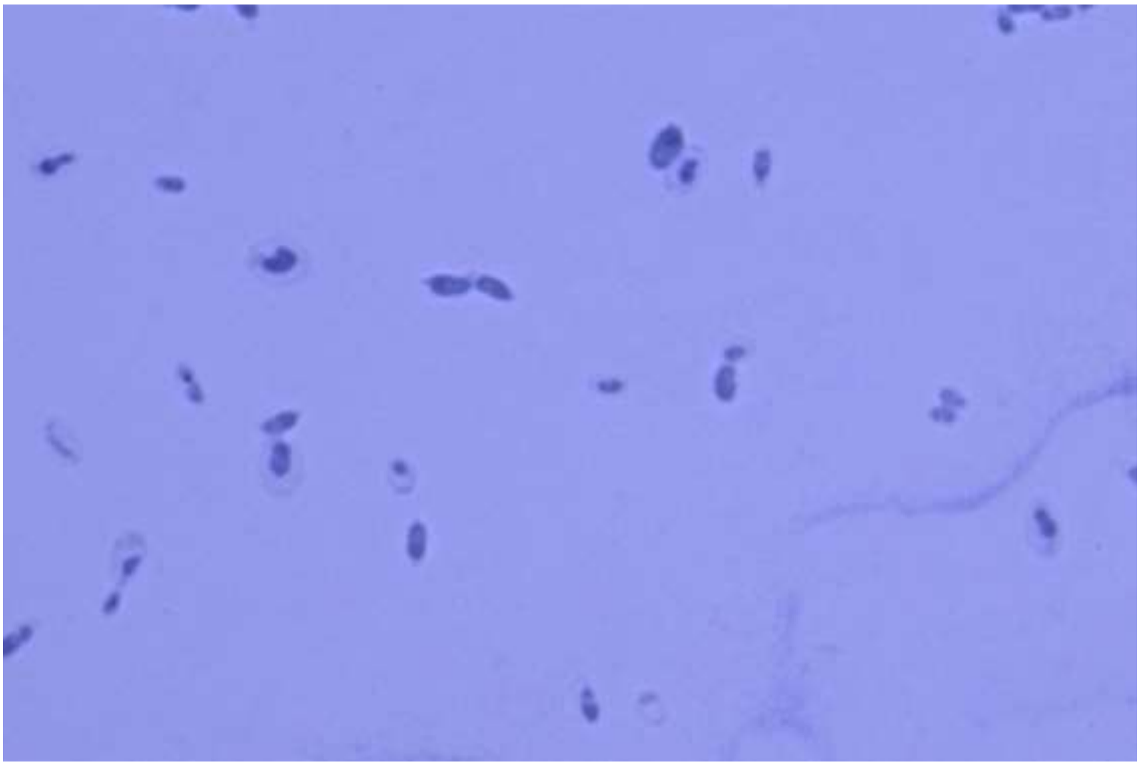

Candida tropicalis with 4-terpinen-ol

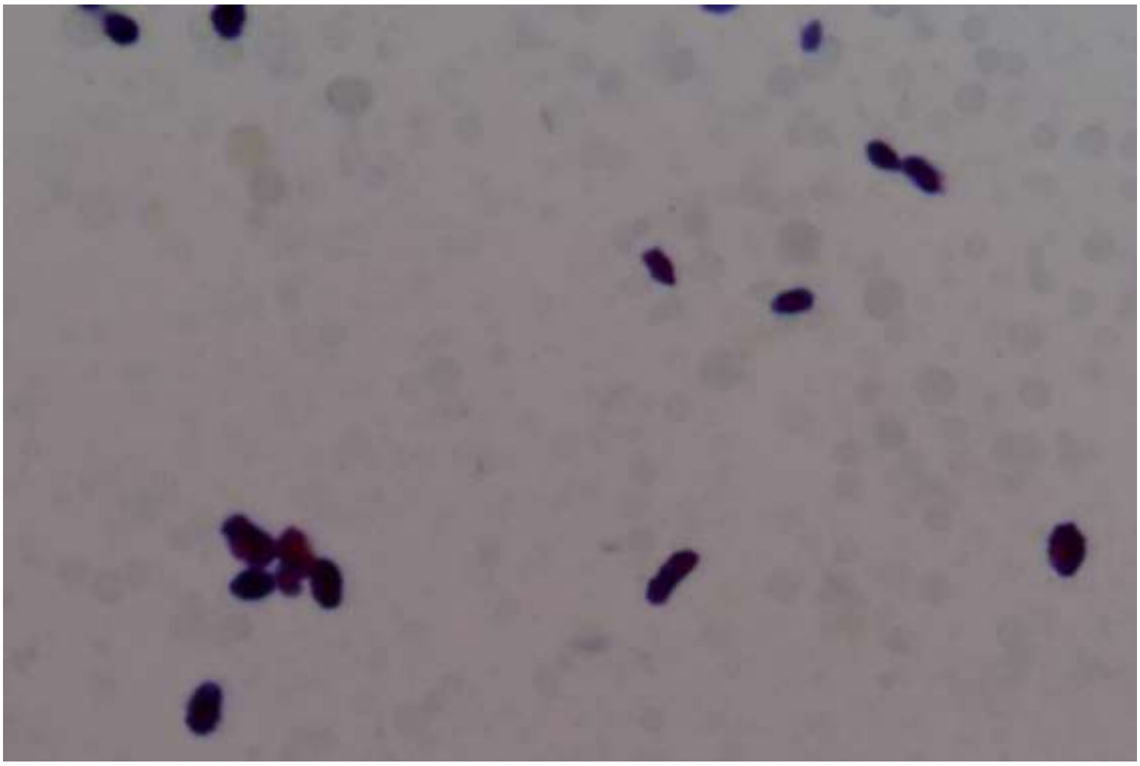

Candida tropicalis with thymol

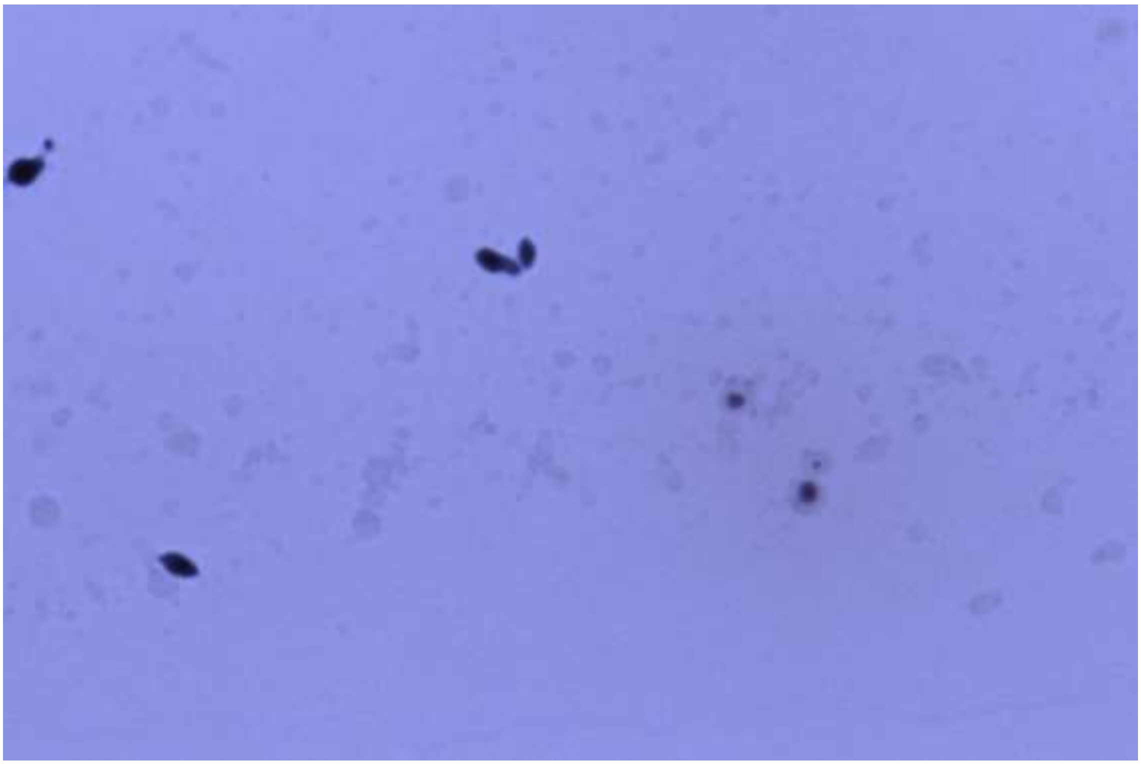

Supplement: Supplementary file 1 [file antibiotics-14-01202-s001.zip › supplementary material 2.pdf]
